# How Does Prior Knowledge Influence Learning Engagement? The Mediating Roles of Cognitive Load and Help-Seeking

**DOI:** 10.3389/fpsyg.2020.591203

**Published:** 2020-10-29

**Authors:** Anmei Dong, Morris Siu-Yung Jong, Ronnel B. King

**Affiliations:** ^1^School of Teacher Education, Guangdong University of Education, Guangzhou, China; ^2^Faculty of Education, The Chinese University of Hong Kong, Hong Kong, China; ^3^Faculty of Education, University of Macau, Macau, China

**Keywords:** prior knowledge, learning engagement, cognitive load, help-seeking, mediation

## Abstract

Research on learning engagement and cognitive load theory have proceeded in parallel with little cross-over of ideas. The aim of this research was to test an integrative model that examines how prior knowledge influences learning engagement via cognitive load and help-seeking strategies. A sample of 356 students from two middle schools in the north of China participated in the study. Analyses using structural equation modeling revealed that prior knowledge was positively associated with learning engagement, and that this relationship was mediated by cognitive load and instrumental help-seeking. Cognitive load also mediated the impact of prior knowledge on instrumental help-seeking, executive help-seeking and avoidance of help-seeking. The study shows that students with more prior knowledge and lower cognitive load are able to exercise higher levels of instrumental help-seeking, leading to good quality learning engagement. On the other hand, students with less prior knowledge and higher cognitive load are less able to engage in instrumental help-seeking, leading to lower engagement. Based on the research findings, recommendations for how teachers can improve learning engagement through decreasing cognitive load are discussed.

## Introduction

Learning engagement is an important educational outcome for twenty-first century students (Jong et al., 2013; Fullan et al., 2018). It is a core indicator of learning processes and outcomes and is an optimal target for educational research given its malleability (Fredricks et al., 2004; Lawson and Lawson, 2013). Past studies on engagement, however, have mostly focused on the critical role of task characteristics, motivational constructs, social factors (e.g., teachers and peers), and self-regulated learning (Hughes et al., 2008; Furrer et al., 2014; Jong et al., 2019). Scant attention has been paid to the role of prior knowledge. In fact, many of the empirical studies on engagement fail to account for prior knowledge or baseline achievement as engagement is usually posited as a predictor of achievement (Martin and Dowson, 2009; Froiland and Oros, 2014; Fung et al., 2018).

This is an important gap because prior knowledge has been shown to be an important factor in the learning process according to cognitive load theory (Bartlett., 1995; Yeh et al., 2012). Prior knowledge helps to decrease cognitive load leading to good learning performance (Myhill and Brackley, 2004; Mihalca et al., 2011; van Riesen et al., 2019). As Shapiro (2004) noted, prior knowledge interacts with other variables to influence learning outcomes. The relationship between prior knowledge and learning engagement can be further enhanced by self-regulated learning (Yang et al., 2018). Help-seeking is a core self-regulated learning strategy (Ryan et al., 2005). However, research on learning engagement on the one hand and cognitive load theory and self-regulated learning research, on the other hand, have proceeded in parallel with little cross-over of ideas. Hence, the aim of this study is to test a model that attempts to identify the theoretical mechanisms through which prior knowledge predicts engagement by drawing on research from cognitive load theory and self-regulated learning theory. More specifically, we tested whether prior knowledge predicted engagement via cognitive load and help-seeking strategies.

This study would advance our understanding of learning engagement in three ways. First, the study would examine an integrative theoretical model combining variables derived from cognitive load theory and self-regulated learning theory. Second, the study would provide evidence for how prior knowledge influences learning engagement.

### Learning Engagement

learning engagement is viewed as a multifaceted construct with three dimensions: behavioral engagement, the attention and effort that students put into learning activities or tasks (Kong et al., 2003; Fredricks et al., 2004); cognitive engagement, which focuses on learning strategies and self-regulation (Pintrich and De Groot, 1990; Fredricks et al., 2004); and emotional engagement, the level of interest in learning (Kong et al., 2003; Fredricks et al., 2004; Skinner and Pitzer, 2012).

Learning something new is predicated on what one already knows (i.e., prior knowledge). Past studies claimed that prior knowledge has an important influence on learning engagement (Rodrigues, 2007; Pecore et al., 2017). Prior knowledge could reduce cognitive load leading to better learning engagement (van Riesen et al., 2019). Yang et al. (2018) claimed self-regulated learning enhanced the relationship between prior knowledge and learning. Therefore, when assessing the influence of prior knowledge on learning engagement, we should consider prior knowledge as a variable that interacts with both cognitive load and help obtained through self-regulated learning to affect learning engagement.

### Prior Knowledge and Cognitive Load Theory

Cognitive load theory states that when acquiring new knowledge, novel information is processed in the working memory, which has a limited capacity, and a new cognitive schema is constructed in the long-term memory (Sweller et al., 1998). Cognitive load negatively influences engagement (Kirschner et al., 2011), self-regulated learning (Hughes et al., 2018), and achievement (Leppink et al., 2014; Chang, 2018).

Paas et al. (1994) listed the influential factors of cognitive load as subject characteristics, task characteristics and subject–task interactions. Prior knowledge is a subject characteristic, and students with more prior knowledge may have more working memory capacity available to process their current learning tasks (Mihalca et al., 2011). According to schema theory, prior knowledge is a critical factor in forming a new cognitive schema to gain new knowledge (Bartlett., 1995). Prior knowledge decrease cognitive load leading to good learning engagement (Myhill and Brackley, 2004; Mihalca et al., 2011). Students with low prior knowledge need more assistance to decrease cognitive load, while those with high prior knowledge more easily form new schema and perceive a lower cognitive load (Myhill and Brackley, 2004; van Riesen et al., 2019).

Cognitive load has a negative relationship with self-regulated learning (Hughes et al., 2018). Both cognitive load and self-regulated learning use up students’ cognitive resources. High cognitive load leads students to choose superficial learning strategies (Galy et al., 2012). Mihalca et al. (2011) assumed that students with a higher level of prior knowledge would have more working memory available to identify their current state of learning and academic needs and be better able to choose their own learning strategy. Prior knowledge influences the effectiveness of help with different cognitive loads. Help provided by instructional support is effective under conditions of low cognitive load with high prior knowledge, but with high cognitive load and low prior knowledge it is ineffective (Seufert et al., 2007).

### Help-Seeking and Self-Regulation

Help-seeking is self-regulation strategy that engages learners’ cognition, behavior and emotions (Puustinen, 1998; Newman, 2000; Karabenick and Berger, 2013). Help-seeking processes reflect the main elements of processes of self-regulation, namely task analysis, strategic planning, self-control, self-judgment and self-reaction (Karabenick and Berger, 2013). When students encounter problems that they cannot solve on their own, they can seek help from teachers and more knowledgeable peers who are able to scaffold them to find or develop solutions (Jong, 2019). Ryan et al. (2005) identified three types of help-seeking behavior: instrumental help-seeking, executive help-seeking, and avoidance of help-seeking. “Instrumental help-seeking” refers to the student’s pursuit of hints and explanations to understand problems, while executive help-seeking is the intention to obtain the answers directly, without understanding them, or to complete a task by depending on others. Avoidance of help-seeking occurs when a struggling student chooses not to seek help.

Help-seeking has been found to be closely related to engagement, achievement, motivation and attitudes toward help-seeking (Ryan and Pintrich, 1997). Instrumental help-seeking has a positive effect on engagement (Marchand and Skinner, 2007; Finney et al., 2018), while executive help-seeking and avoidance of help-seeking have negative effects (Karabenick and Knapp, 1991; Shim et al., 2016). Instrumental help-seeking has a positive effect on achievement, while executive help-seeking and avoidance of help-seeking have negative effects (Ryan et al., 2005; Shim et al., 2016). Hence, different types of help-seeking strategies seem to be differentially linked to learning engagement (Duchesne et al., 2019).

The relationship between prior knowledge and learning engagement can be improved by means of self-regulated learning (Yang et al., 2018). Even more importantly, help provided by instructional support is particularly effective under the conditions of low cognitive load and high prior knowledge (Seufert et al., 2007). Furthermore, prior knowledge strongly influences students’ self-regulation. When prior knowledge is poor, self-regulated learning can enhance learning performance (Yang et al., 2018). Students obtain explanations, which bridge prior knowledge to new concepts, from teachers or peers’ support (Williams and Lombrozo, 2013). In addition, help-seeking behaviors are strategies of self-regulated learning that determine how the quality of help influences learning engagement (Ryan et al., 2005). Therefore, when assessing the influence of prior knowledge on learning engagement, we should consider prior knowledge as a variable that interacts with both cognitive load and help obtained through self-regulated learning to affect learning engagement.

### The Present Study

The aim of the current study was to advance knowledge of the relationship between prior knowledge and learning engagement. Structural equation modeling was used to test the relationships between prior knowledge, cognitive load, help-seeking behaviors, and learning engagement.

De Bruin and Van Merriënboer (2017) argued that the cue utilization framework (Koriat, 1997) can bridge cognitive load and self-regulated learning research in one integrated framework. Our study is broadly informed by this framework. The integrated framework includes a model with three elements: cues, cognitive load ratings/regulatory learning and actual learning performance. The integrated model is as follows: instructional strategies help learners to recognize cues which are utilized for cognitive load rating and regulation judgment; cues are utilized for diagnosing actual learning performance; and cognitive load rating and regulation judgment lead to higher learning and performance by improving the regulation of learning activities and apportioning of mental resources.

The hypothesized research model for the present study, based on De Bruin’s framework, is as follows. First, help-seeking is a strategy of self-regulated learning in the model. We aim to test the effectiveness of different types of help-seeking behavior, namely instrumental help-seeking, executive help-seeking and avoidance of help-seeking. Second, prior knowledge, as a prerequisite for learning, is the cue for students to construct a new schema and rate their cognitive load, make judgments about which self-regulated learning strategies they will choose and diagnose learning engagement. Third, cognitive load and the three types of help-seeking behavior influence learning engagement. According to Seufert et al. (2007), we assume that cognitive load mediates the impact of prior knowledge on help-seeking behaviors.

**FIGURE 1:**
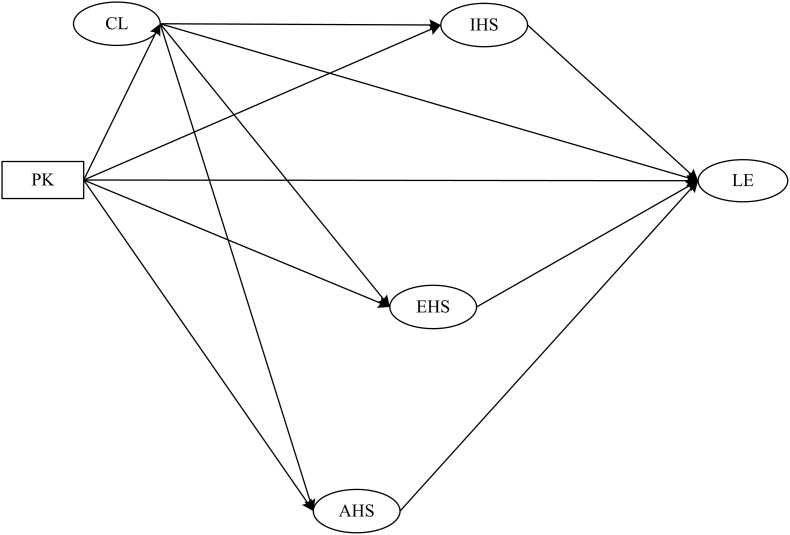
Hypothesized research model (PK, prior knowledge; CL, cognitive load; IHS, instrumental help-seeking; EHS, executive help-seeking; AHS, avoidance of help-seeking; LE, learning engagement).

The hypothesized model is shown in Figure 1. Structural equation modeling was utilized to explore the relationships between prior knowledge, cognitive load, three types of help-seeking behavior and learning engagement. Prior knowledge was treated as an exogenous variable, and cognitive load, instrumental help-seeking, executive help-seeking, avoidance of help-seeking and learning engagement were treated as endogenous latent variables.

The following hypotheses were proposed: *H1*: Prior knowledge has a positive effect on learning engagement. *H2*: Prior knowledge has a negative effect on cognitive load. *H3a:* Cognitive load has a negative effect on instrumental help-seeking. *H3b:* Cognitive load has a positive effect on executive help-seeking. *H3c:* Cognitive load has a positive effect on avoidance of help-seeking. *H4*: Cognitive load mediates the impact of prior knowledge on instrumental help-seeking, executive help-seeking, and avoidance of help-seeking. *H5*: Cognitive load, instrumental help-seeking, executive help-seeking, and avoidance of help-seeking mediate the impact of prior knowledge on learning engagement.

## Materials and Methods

### Participants and Procedure

The participants were 356 secondary school students (48.9% male and 51.1% female) from two schools in the north of China. They were aged 12 (7.6%), 13 (61.2%), 14 (29.8%), and 15 (1.4%). The two schools were average-performing public schools, and the principals at each school had expressed the desire to promote teaching quality and learning performance. The data were collected in two stages. First, midterm exam scores were collected from the academic affairs division of each school. Then, at the end of the term, questionnaire data were collected. Both sets of data included the students’ names and ID numbers, which were used to match the data from each set. The researchers did not conduct any interventions in the schools, and the teachers implemented their teaching as usual.

The questionnaires and study plan were reviewed and approved by the research ethics committee of the Chinese University of Hong Kong before the survey was conducted. Permission and informed consent were obtained from the two schools’ principals and head teachers. All students’ participation was authorized by the students’ parents. To minimize the social expectation effect, the researchers told the class teachers and students that the data would be used only for research purposes and would have no influence on their achievement or studies. Regarding the instructions concerning how to fill in the questionnaire, all items related to math classrooms and learning, and details of the sample items were read aloud to the participants in the administering process. The participants were told to provide true answers to each question, for which there was no right or wrong response, and that the research results would help to improve future teaching quality.

### Instruments

#### Learning Engagement Questionnaire

The Learning Engagement Questionnaire comprises 30 items related to the behavioral, cognitive, and emotional dimensions of learning engagement. The behavioral and emotional engagement dimensions were adopted from Kong et al. (2003), who developed the instrument in a Chinese math class. Behavioral engagement consisted of attention and effort, which were each indexed by six items. A sample item is, “I listen to the teacher’s instruction attentively.” Emotional engagement consisted of interest, which was indexed by six items. A sample item is, “I am always curious to learn new things in math and I find learning math enjoyable.” The cognitive engagement dimension was adapted from Pintrich and De Groot (1990) and Pintrich et al. (1993), which have been shown to have construct validity in the Chinese version (Rao and Sachs, 1999; Tong et al., 2019). It consisted of regulation, monitoring, planning and organization, which were indexed by three items each. A sample item is, “When studying for this course I try to determine which concepts I don’t understand well.”

Learning engagement is an endogenous latent variable with three indicator variables, namely behavioral engagement, cognitive engagement and emotional engagement. The value of behavioral engagement was the mean of attention and effort, derived from the means of their six items. The value of cognitive engagement was the mean of regulation, monitoring, planning and organization, derived from the means of their three items. The value of emotional engagement was the mean of interest (six items). After considering the feedback from interviews with teachers and students in the two schools, rehearsal and elaboration were not included in the cognitive engagement dimension. The teachers required the students to use mathematics terms and not use their own words to express concepts; thus, “elaboration” was not appropriate for this study. As the teachers expected students to understand the mathematics concepts or formulae but not recite them, “rehearsal” was also not appropriate for this study.

#### Cognitive Load Questionnaire

The Cognitive Load Questionnaire was developed by Hwang et al. (2013) based on the measures proposed by Paas et al. (1994) and Sweller et al. (1998). A Chinese version of the Cognitive Load Questionnaire been used in Chinese middle school classrooms (Wang et al., 2018). The Cognitive Load Questionnaire consists of five items for mental load and three items for mental effort. Sample items include, “The learning content in this learning activity was difficult for me” (mental load) and “I need to put lots of effort into completing the learning tasks or achieving the learning objectives in this learning activity” (mental effort).

#### Help-Seeking Questionnaire

The Help-Seeking Questionnaire, adopted from the Chinese version of the questionnaire (Li, 1999), comprised 13 items describing the 3 types of help-seeking behavior. It included five items for instrumental help-seeking, four for executive help-seeking and four for avoidance of help-seeking. The items for instrumental help-seeking and avoidance of help-seeking were adapted from Ryan and Pintrich (1997) by Li (1999), and executive help-seeking was developed by Li (1999) based on Nelsonle-Le Gall (1985) and Karabenick and Knapp (1991) for the Chinese classroom context. Sample items include, “If I get stuck on a math problem, I ask someone for help so I can keep working on it” (instrumental help-seeking), “For math problems, I ask for the right answers without trying” (executive help-seeking), and “I don’t ask for help in math, even if the work is too hard to solve on my own” (avoidance of help-seeking). To test for sex invariance in help-seeking, multi-group confirmatory factor analysis in Mplus was used to test the hypothesis that the six models set by factorial invariance were different. Chi-square tests of comparison models were not significant and differences in the TLI (Tucker-Lewis index) and CFI (comparative fit index) were less than 0.01, indicating that there were no significant differences in the six models. These results demonstrate that help-seeking behavior has the same meaning and potential structure among male and female students.

#### Prior Knowledge

Students’ math prior knowledge was indexed by their mid-term scores on tests developed by math teachers in their school according to the Compulsory Education Mathematics Curriculum Standards of the Chinese Ministry of Education. The test questions were not the same in School A and School B because they used different textbooks. Mid-term tests in both schools included 10 one-choice questions, 8 fill-in-the-blanks questions, and 5 solution questions with total scores of 30, 24, and 66. The tests aimed to evaluate the students’ relevant math knowledge and ability.

All of the items were reviewed by two middle school teachers, who revised some wording to make the scale easier for middle school students to understand. Finally, after revision and comparison with the original English items, all of the Chinese items were agreed upon by the research team.

The participants responded to each questionnaire item on a scale from 1 (extremely unlikely) to 6 (extremely likely). On the 6-point scale, “1” indicated the lowest level of learning engagement, help-seeking (instrumental help-seeking, executive help-seeking, avoidance of help-seeking) and cognitive load. The completed questionnaires were collected from the participants in the classroom at the end of the term. To measure prior knowledge, math test results were collected at mid-term. A perfect score on the test was 120.

### Data Analysis

SPSS 21 and Mplus 7 for Windows were used to assist with the data analysis. We conducted the analyses in four steps. First, a missing value analysis was carried out to examine patterns in the missing responses. The result showed that missing values were less than 5% for every variable; thus, an expectation maximization algorithm was used to handle missing data in the analysis. Second, descriptive statistics (M, SD, skewness, and kurtosis) and correlations were calculated. Cronbach’s α coefficients were used to examine the subscales’ reliability. Third, we used confirmatory factor analysis (CFA) to detect the validity of the three constructs, including between-item relationships, latent variables and fit indices. Fourth, we used structural equation modeling to examine the direct and mediated effects between the variables. The Maximum Likelihood Robust (MLR) estimator was used in Mplus to avoid bias in the estimates of parameters and standard errors. There were two considerations: (1) two univariate variables were non-normal; and (2) although absolute values of skewness and kurtosis of variables in the structural model met the assumption of the multivariate normal distribution for structural equation modeling, the data sample was smaller than the cutoff of 400 required for valid MLR estimation (Yuan and Bentler, 1998).

## Results

### Descriptive Statistics and Correlations

Table 1 presents the descriptive results for the constructs and items, including the mean, standard deviation, skewness, kurtosis, and Cronbach’s alpha. The mean of prior knowledge is mid-term scores. The mean of cognitive load is the mean of mental load and mental effort. The means of instrumental help-seeking, executive help-seeking, and avoidance of help-seeking are the means of their respective items. The mean of learning engagement is the mean of behavioral engagement, cognitive engagement and emotional engagement, as previously calculated in the literature (e.g., Marchand and Furrer, 2014; Reeve and Lee, 2014).

**TABLE 1:** Descriptive statistics (*n* = 356).

	Mean	SD	Skewness	Kurtosis	Cronbach’s α
1. PK	85.70	26.35	−0.738	−0.276	–
2. CL	2.95	1.10	0.352	−0.338	0.86
3. IHS	3.88	1.30	−0.200	−0.800	0.86
4. EHS	1.85	0.89	1.475	3.096	0.67
5. AHS	2.21	1.07	0.999	0.774	0.73
6. LE	4.38	0.96	−0.491	−0.109	0.95

*PK, prior knowledge; CL, cognitive load; IHS, instrumental help-seeking; EHS, executive help-seeking; AHS, avoidance of help-seeking; LE, learning engagement.*

The absolute values of skewness ranged from 0.200 to 1.475, and kurtosis ranged from 0.077 to 3.096. Cronbach’s alpha was used to assess the internal consistency of the scales. As reported in Table 1, most of the constructs returned Cronbach’s alpha values above 0.7, indicating acceptable internal consistency. The Cronbach’s alpha values of the help-seeking behavior scales in Tanaka et al. (2001) were 0.67 and 0.56 for instrumental help-seeking and avoidance of help-seeking, respectively, and Finney et al. (2018) reported values of 0.34, 0.68, and 0.71 for instrumental help-seeking, executive help-seeking, and avoidance of help-seeking, respectively. Thus, the Cronbach’s alpha of 0.67 for executive help-seeking in this study was deemed to be acceptable based on prior literature^1^.

Table 2 shows the correlations between the variables. All were significant at the alpha level of 0.01 or 0.05, and below the threshold of 0.8. This indicates no serious problem of multicollinearity.

**TABLE 2:** Correlation matrix.

	1	2	3	4	5	6
1. PK	1					
2. CL	−0.424**	1				
3. IHS	0.129*	−0.129**	1			
4. EHS	−0.358**	0.576**	−0.152**	1		
5. AHS	−0.314**	0.545**	−0.395**	0.605**	1	
6. LE	0.383**	−0.437**	0.528**	−0.444**	−0.529**	1

*Correlation is significant at the 0.05 level (2-tailed).**Correlation is significant at the 0.01 level (2-tailed).PK, prior knowledge; CL, cognitive load; IHS, instrumental help-seeking; EHS, executive help-seeking; AHS, avoidance of help-seeking; LE, learning engagement.

### Structural Model

First, we utilized CFA to examine the validity of the four constructs of cognitive load, instrumental help-seeking, executive help-seeking, avoidance of help-seeking and learning engagement. The chi-square test is sensitive to sample size and is suitable for a sample of 100–200 (Rigdon, 1995). Therefore, to assess the goodness of fit of the constructs and the model, we used indices, including the comparative fit index (CFI), the Tucker-Lewis index (TLI) and the standardized root mean square residual index (RMSEA). Table 3 shows the goodness of fit indices for the four constructs, which were found to be acceptable to develop a structural model. The results demonstrate an acceptable fit with the data, indicating adequate validity for all of the factors in the structural model (CFI = 0.942, TLI = 0.928, RMSEA = 0.052).

**TABLE 3:** Model fit indices.

Construct	^χ 2^	*df*	^χ 2^*/df*	CFI	TLI	RMSEA [90% CI]	*p*
CL	41.052	19	2.41	0.968	0.953	0.058 [0.034, 0.083]	0.002
IHS	10.050	5	2.01	0.988	0.977	0.054 [0.000, 0.094]	0.074
EHS	4.731	2	2.37	0.975	0.924	0.063 [0.000, 0.139]	0.094
AHS	4.121	2	2.06	0.985	956	0.056 [0.000, 0.133]	0.127
LE	116.849	51	2.29	0.964	0.953	0.061 [0.047, 0.076]	0.000
Structural model	246.839	138	1.79	0.942	0.928	0.052 [0.042, 0.061]	0.000
Recommended values			<3	>0.90	>0.90	<0.08	>0.05

*PK, prior knowledge; CL, cognitive load; IHS, instrumental help-seeking; EHS, executive help-seeking; AHS, avoidance of help-seeking; LE, learning engagement.*

### Hypothesis Testing

#### Direct Paths

We used structural equation modeling with the robust estimator (MLR) to test direct and indirect relationships. All of the items were significant at a 0.01 level on their latent factors and all of the factor loadings ranged between 0.53 and 0.93.

**FIGURE 2:**
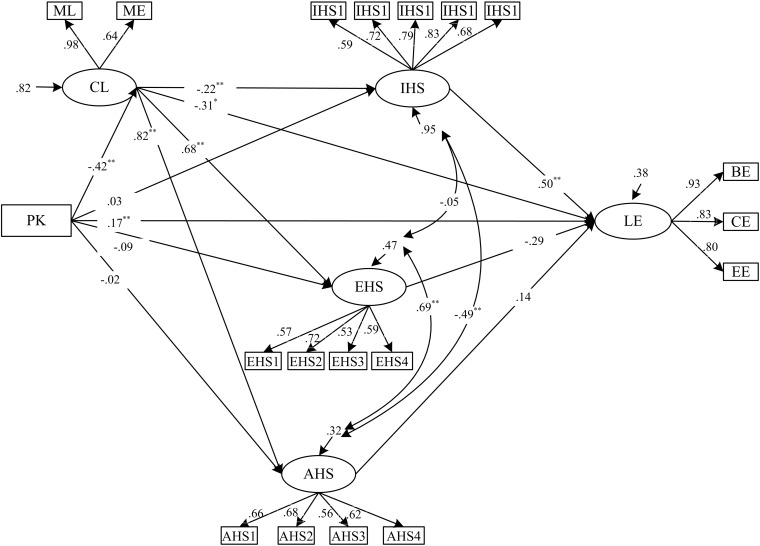
Structural equation modeling results of the structural model with standardized coefficients (PK, prior knowledge; CL, cognitive load; IHS, instrumental help-seeking; EHS, executive help-seeking; AHS, avoidance of help-seeking; LE, learning engagement). *p < 0.05, **p < 0.01.

Figure 2 shows the results of analyses of the proposed relationships between the six variables: prior knowledge, cognitive load, instrumental help-seeking, executive help-seeking, avoidance of help-seeking and learning engagement. H1, H2, H3a, H3b, and H3c are supported. Prior knowledge has a significant positive direct effect on engagement (β = 0.17, *p* < 0.01) (H1), a finding that is aligned with the results from previous studies (Rodrigues, 2007; Pecore et al., 2017). Consistent with findings of van Riesen et al. (2019), prior knowledge has a significant negative direct effect on cognitive load (β = − 0.42, *p* < 0.01) (H2). Cognitive load has a significant negative direct effect on instrumental help-seeking (β = − 0.22, *p* < 0.01) (H3a); and cognitive load has significant positive direct effects on executive help-seeking (β = 0.68, *p* < 0.01) and avoidance of help-seeking (β = 0.82, *p* < 0.01) (H3b, H3c); these results demonstrate the specific relationships between the variables, in alignment with the findings of Hughes et al. (2018).

#### Indirect Paths

The total effect of prior knowledge on learning engagement was 0.42, while the direct effect of prior knowledge on learning engagement was 0.17 and the indirect effect of prior knowledge on learning engagement was 0.25. The mediating effects accounted for 59.5% (0.25/0.42) of the total. Therefore, indirect effects play an important role in explaining the mechanism between prior knowledge and learning engagement. The indirect effect of prior knowledge on learning engagement is the sum of paths 1a, 1b, 1c, 1d, 1e, 1f, and 1g (see the standardized beta weights in Table 4).

**TABLE 4:** Standardized coefficients for direct and indirect effects of PK on LE through CL, IHS, EHS, and AHS.

	β	*p*	95% CI
*Direct path*			
1. PK→LE	0.169**	0.001	[0.087, 0.250]
2. PK→IHS	0.030	0.628	[−0.072, 0.132]
3. PK→EHS	−0.094	0.187	[−0.212, 0.023]
4. PK→AHS	−0.020	0.745	[−0.121, 0.081]
5. CL→LE	−0.314*	0.028	[−0.551,−0.078]
*Indirect path*			
**1. PK→LE**	0.252*	**0.000**	**[0.176, 0.327]**
**1a. PK→CL→LE**	0.132*	**0.032**	**[0.031, 0.233]**
1b. PK→IHS→LE	0.015	0.630	[−0.037, 0.067]
1c. PK→EHS→LE	0.027	0.455	[−0.033, 0.087]
1d. PK→AHS→LE	−0.003	0.814	[−0.022, 0.017]
**1e. PK→CL→IHS→LE**	0.046*	**0.021**	**[0.013, 0.078]**
1f. PK→CL→EHS→LE	0.082	0.306	[−0.050, 0.214]
1g. PK→CL→AHS→LE	−0.048	0.725	[−0.269, 0.174]
**2. PK→CL→IHS**	0.091**	**0.007**	**[0.035, 0.146]**
**3. PK→CL→EHS**	−0.284**	**0.000**	**[−0.364,−0.204]**
**4. PK→CL→AHS**	−0.342**	**0.000**	**[−0.431,−0.252]**
5. CL→LE	−0.191	0.193	
**5a. CL→IHS→LE**	−0.109*	**0.019**	**[−0.185,−0.032]**
5b. CL→EHS→LE	−0.195	0.309	[−0.511, 0.121]
5c. CL→AHS→LE	0.113	0.725	[−0.417, 0.643]

*p < 0.05, **p < 0.01. PK, prior knowledge; CL, cognitive load; IHS, instrumental help-seeking; EHS, executive help-seeking; AHS, avoidance of help-seeking; LE, learning engagement. The significant indirect paths are showed in bold.

As shown by the indirect effect of prior knowledge on learning engagement, cognitive load mediates the impact of prior knowledge on learning engagement (Path 1a: β = 0.13, *p* < 0.05) and the mediating effects account for 31% (0.13/0.42) of the total effect of prior knowledge on learning engagement. Cognitive load and instrumental help-seeking jointly mediate the effect of prior knowledge on learning engagement (Path 1e: β = 0.05, *p* < 0.05) and the mediating effects account for 12% (0.05/0.42) of the total effect of prior knowledge on learning engagement. Other decomposition paths of this indirect effect are not significant. Therefore, H5 is partly supported, largely consistent with the findings of Seufert et al. (2007).

From Paths 2, 3, and 4, we can see that cognitive load mediates the impact of prior knowledge on instrumental help-seeking (β = 0.09, *p* < 0.01), executive help-seeking (β = −0.28, *p* < 0.01) and avoidance of help-seeking (β = −0.34, *p* < 0.01). The mediating effects account for 75% [0.09/(0.03 + 0.09)], 76%[−0.0.28/(−0.09 −0.28)], and 94%[−0.0.34/(−0.02 − 0.34)] of the total effect of prior knowledge on instrumental help-seeking, executive help-seeking and avoidance of help-seeking, respectively. Therefore, H4 is supported, based on the research of Myhill and Brackley (2004).

From Path 5a, we can see that instrumental help-seeking mediates the impact of cognitive load on learning engagement (β = −0.11, *p* < 0.05). The mediating effects account for 22%[−0.0.11/(−0.19 −0.31)] of the total effect of cognitive load on prior knowledge.

## Discussion

Grounded on De Bruin and Van Merriënboer (2017) framework, this study explores the effects of prior knowledge on learning engagement mediated by cognitive load and help-seeking behaviors, bridging cognitive load and self-regulated learning research. It puts concrete self-regulated learning strategies, namely help-seeking behaviors (instrumental help-seeking, executive help-seeking, and avoidance of help-seeking) into the research model.

Prior knowledge had a positive effect on learning engagement, which is consistent with claims made by other researchers (Rodrigues, 2007; Pecore et al., 2017). However, our work further explores the direct and indirect effects. Significant mediated effects accounted for 43% of the total effect. First, cognitive load as a single mediated variable between prior knowledge and learning engagement accounted for 31% of the total effect. More prior knowledge gives students more working memory to acquire more new knowledge to enhance their learning engagement (Sweller et al., 1998). Cognitive load is also affected by instructional design, which can reduce extraneous cognitive load or increase germane cognitive load (Kirschner et al., 2011). Therefore, the effect of the level of students’ prior knowledge could have been influenced by external instructional design that accommodated cognitive load to promote engagement in learning. If teachers take advantage of technology to explain complicated concept and assign small unit task in learning, students will have lower level cognitive load and lead to better learning engagement.

Second, cognitive load and instrumental help-seeking jointly mediated the relationship between prior knowledge and learning engagement, which accounted for 12% of the total effect of prior knowledge on learning engagement. Jointly, cognitive load and executive help-seeking/avoidance of help-seeking did not mediate the impact of prior knowledge on learning engagement. Consistent with the study of Shapiro (2004), prior knowledge interacted with cognitive load and instrumental help-seeking to affect learning engagement. Executive help-seeking and avoidance of help-seeking do not promote understanding knowledge, but students update their cognitive schema with instrumental help-seeking (Ryan et al., 2005). Therefore, students who have a higher level of prior knowledge and lower cognitive load may be able to allocate cognitive resources to instrumental help-seeking to enhance their understanding, leading to good quality learning engagement. If teachers design simple tasks for students who have lower level of prior knowledge could choose instrumental help-seeking, students would benefit from learning process and engage more in learning.

Third, cognitive load had a negative effect on instrumental help-seeking, but positive effects on executive help-seeking and avoidance of help-seeking due to the opposite nature of these help-seeking behaviors. Intuitively, we expect that students perceiving a high cognitive load might engage in instrumental help-seeking to lessen the load. However, when students experience a high cognitive load, they are unable to manage their learning due to limited working memory and cognitive recourses.

Fourth, cognitive load mediated the impact of prior knowledge on instrumental help-seeking, executive help-seeking, and avoidance of help-seeking. Although we explain cognitive load as a mediating variable between prior knowledge and learning engagement, the mediated effects will aggravate different help-seeking behaviors that affect student learning. The mediating effects accounted for 75, 76, and 94% of the total effect of prior knowledge on instrumental help-seeking, executive help-seeking, and avoidance of help-seeking, respectively. These are very high percentages, indicating mediated effects that play key roles in the relationships between prior knowledge and the three types of help-seeking. As Amadieu et al. (2009) argued, different levels of prior knowledge result in different outcomes with less structured help, but have the same outcome with well-structured help. We therefore infer that students can adopt instrumental help-seeking to bring about good quality support for their learning. If teachers expect students to use instrumental help-seeking to improve learning, decreasing their cognitive load is a very important way to ensure that students have available cognitive resources to handle instrumental help-seeking. Furthermore, instrumental help-seeking mediates the impact of cognitive load on learning engagement. By the same rationale, the degree of cognitive load influences learning engagement through available cognitive recourses and students obtain real help through instrumental help-seeking, which helps them to construct a cognitive schema to improve learning engagement.

Despite its strengths, this study also has limitations. First, causal relations cannot be established as we relied on cross-sectional approaches. Experimental studies are needed to make causal conclusions. Second, the study did not measure objective performance and only relied on self-reported engagement. This might have led to common method bias. Future studies may want to incorporate objective measures of engagement or achievement. Third, the social aspects of learning have not been included. Future research could examine how students interact with their peers and teachers as these are usually the people from whom they seek help from.

## Conclusion

Learning engagement is strongly influenced by prior knowledge. However, past studies on engagement have failed to take this into account. Our findings indicate that cognitive load plays a crucial role in the relationship between prior knowledge and learning engagement via help-seeking behaviors. Paradoxically, it is students who least need help because they already know more (high prior knowledge) are also more likely to engage in adaptive instrumental help-seeking. Conversely, students who most needed help because they knew less (low prior knowledge) were less likely to seek help or seeking or engage in executive help-seeking. A practical implication of this study is that teachers should pay attention to adjusting the level of cognitive load through their instructional design to facilitate students’ instrumental help-seeking thereby promoting learning engagement.

## Data Availability Statement

The raw data supporting the conclusions of this article will be made available by the authors, without undue reservation.

## Ethics Statement

The studies involving human participants were reviewed and approved by the Survey and Behavioural Research Ethics Committee of the Chinese University of Hong Kong. Written informed consent to participate in this study was provided by the participants’ legal guardian/next of kin.

## Author Contributions

All authors listed have made a substantial, direct and intellectual contribution to the work, and approved it for publication.

## Conflict of Interest

The authors declare that the research was conducted in the absence of any commercial or financial relationships that could be construed as a potential conflict of interest.

## Footnotes

1 When executive help-seeking, for which Cronbach’s alpha is lower than 0.7, is not included in the model, the model fit indices (CFI = 0.955, TLI = 0.945, RMSEA = 0.048) are better than those reported in the paper. However, if executive help-seeking is excluded from the model, the mediating effect between prior knowledge, cognitive load, executive help-seeking is not visible. In addition, the *p*-value of the direct effect of cognitive load on learning engagement is *p* = 0.008 < 0.01, whereas in this paper, it is *p* = 0.022 < 0.05. However, despite these minor differences, the substantive results of our paper remain. Support for our core hypotheses did not change whether or not we included executive help-seeking. This supports the robustness of our results (The results of this alternative model are attached in the Supplementary Material).
