# Supplementary material for: How Does Prior Knowledge Influence Learning Engagement? The Mediating Roles of Cognitive Load and Help-Seeking
Source: Front Psychol. 2020 Oct 29;11:591203. doi: 10.3389/fpsyg.2020.591203 (PMC7658369; doi:10.3389/fpsyg.2020.591203)
Supplement: Supplementary file 1 [file Data_Sheet_1.pdf]

Results of the alternative model without executive help-seeking and mental effort:

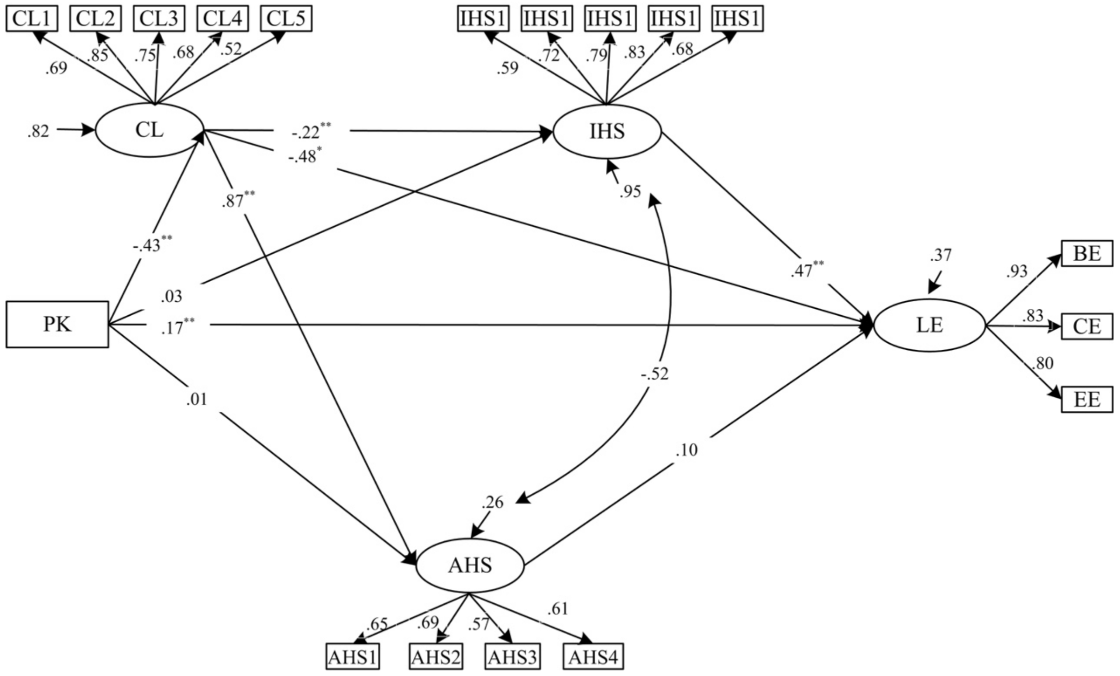

FIGURE 2 | SEM results of the alternative structural model with standardized coefficients. (PK, prior knowledge; CL, cognitive load; IHS, instrumental help-seeking; AHS, avoidance of help-seeking, LE, learning engagement). $^{*}p < 0.05$, $^{**}p < 0.01$.

(1) The five manifest variables of CL were items of mental load.

(2) This model does not include EHS (executive help-seeking).

Table 3 | Model fit indices.

Construct	χ^2	*df*	χ^2/df	CFI	TLI	RMSEA [90% CI]	*p*
CL	7.459	5	1.49	0.994	0.987	0.038 [0.000, 0.090]	0.189
IHS	10.050	5	2.01	0.988	0.977	0.054 [0.000, 0.094]	0.074
AHS	4.121	2	2.06	0.985	0.956	0.056 [0.000, 0.133]	0.127
LE	116.849	51	2.29	0.964	0.953	0.061 [0.047, 0.076]	0.000
Structural model	226.216	126	1.79	0.955	0.945	0.048 [0.038, 0.058]	0.000
Recommended values			<3	>0.90	>0.90	<0.08	>0.05

PK, prior knowledge; CL, cognitive load; IHS, instrumental help-seeking; EHS, executive help-seeking; AHS, avoidance of help-seeking; LE, learning engagement.

Table 4 | Standardized coefficients for direct and indirect effects of PK on LE through CL, IHS, and AHS

	β	*p*	95% CI
Direct path			
1. PK→LE	0.170**	0.001	[0.091, 0.248]
2. PK→IHS	0.025	0.699	[-0.080, 0.130]
4. PK→AHS	0.014	0.796	[-0.074, 0.102]
5. CL→LE	-0.457**	0.008	[-0.741, -0.173]
Indirect path			
**1. PK→LE**	**0.249***	**0.000**	**[0.178, 0.320]**
**1a. PK→CL→LE**	**0.196***	**0.011**	**[0.069, 0.324]**
1b. PK→IHS→LE	0.012	0.700	[-0.038, 0.061]
1d. PK→AHS→LE	0.000	0.963	[-0.005,0 .005]
**1e. PK→CL→IHS→LE**	**0.045****	**0.016**	**[0.014, 0.075]**
1g. PK→CL→AHS→LE	-0.004	0.962	[-0.128, 0.121]
**2. PK→CL→IHS**	**0.096****	**0.011**	**[0.033, 0.158]**
**4. PK→CL→AHS**	**-0.372****	**0.000**	**[-0.462, -0.282]**
5. CL→LE	-0.096	0.570	[-0.373, 0.182]
**5a. CL→IHS→LE**	**-0.104**	**0.011**	**[-0.172, -0.037]**
5c. CL→AHS→LE	0.008	0.962	[-0.281, 0.297]

$^{*}p < 0.05$, $^{**}p < 0.01$. PK, prior knowledge; CL, cognitive load; IHS, instrumental help-seeking; EHS, executive help-seeking; AHS, avoidance of help-seeking; LE, learning engagement.

The significant indirect paths are showed in bold.
